# Multimorbidity, functional impairment, and mortality in older patients stable after prior acute myocardial infarction: Insights from the TIGRIS registry

**DOI:** 10.1002/clc.23915

**Published:** 2022-11-01

**Authors:** Akshay Bagai, Faeez M. Ali, John Gregson, Karen P. Alexander, Mauricio G. Cohen, Karolina Andersson Sundell, Tabassome Simon, Dirk Westermann, Satoshi Yasuda, David Brieger, Shaun G. Goodman, Jose C. Nicolau, Christopher B. Granger, Stuart Pocock

**Affiliations:** ^1^ Terrence Donnelly Heart Center, St. Michael's Hospital University of Toronto Toronto Ontario Canada; ^2^ Department of Medical Statistics London School of Hygiene and Tropical Medicine London UK; ^3^ Duke Clinical Research Institute, Duke University School of Medicine Durham North Carolina USA; ^4^ University of Miami Miller School of Medicine Miami Florida USA; ^5^ Biopharmaceuticals Medical, AstraZeneca Gothenburg Sweden; ^6^ Department of Clinical Pharmacology and Clinical Research Platform of East of Paris Assistance Publique—Hopitaux de Paris Paris France; ^7^ Clinical Pharmacology—Research Platform (UPMC‐Paris 06), Sorbonne Université Paris France; ^8^ Department of Cardiology and Angiology, Medical Center University of Freiburg Freiburg im Breisgau Germany; ^9^ Department of Cardiovascular Medicine Tohoku University Graduate School of Medicine Sendai Japan; ^10^ Cardiology Department Concord Hospital Sydney Australia; ^11^ Department of Medicine, Canadian VIGOUR Centre University of Alberta Edmonton Alberta Canada; ^12^ Instituto do Coracao (InCor), Hospital das Clinicas HCFMUSP, Faculdade de Medicina, Universidade de São Paulo São Paulo Brazil; ^13^ Cardiac Care Unit, Duke University Medical Center Durham North Carolina USA

**Keywords:** Acute coronary syndromes, comorbidities, frailty, functional impairment, multimorbidity, myocardial infarction, older age, outcomes, rehabilitation, stroke

## Abstract

**Background:**

Data on the association of multimorbidity and functional impairment with cardiovascular (CV) and non‐CV outcomes among older myocardial infarction (MI) patients are limited.

**Hypothesis:**

Multimorbidity and functional impairment among older MI patients are associated with CV and non‐CV mortality.

**Methods:**

Patients aged ≥65 years, 1−3 years post‐MI, and enrolled between June 2013 and Novemeber 2014 from 349 sites in 25 countries in the global TIGRIS registry were categorized by age, number of comorbidities, and presence and degree of functional impairment. Functional impairment was calculated using five‐dimension EuroQol based on three domains—mobility, self‐care, and usual activities. The association between age, number of comorbid conditions, and degree of functional impairment with 2‐year incidence of CV and non‐CV death was evaluated using Poisson regression analysis.

**Results:**

Older age was associated with higher number of comorbidities and functional impairment; after adjustment, increasing age was significantly associated with non‐CV mortality (*p* = .03) but not CV mortality (*p* = .38). Greater functional impairment was associated with a higher rate and relatively equal magnitude risk of CV (rate ratios [RR] 1.52, 95% confidence intervals [CI]: 1.29−1.79, per one‐step increase) and non‐CV mortality (RR 1.42, 95% CI: 1.17−1.73). Multimorbidity was more strongly associated with CV mortality (RR 1.52, 95% CI: 1.38−1.67, per additional comorbidity) versus non‐CV mortality (RR 1.29, 95% CI: 1.14−1.47, per additional comorbidity).

**Conclusions:**

Multimorbidity and functional impairment are prevalent among older post‐MI patients and are associated with increased CV and non‐CV mortality. These findings highlight the importance of considering comorbid conditions and functional impairment as predictors of risk for adverse outcomes and aspects of medical decision making. Clinical Trial Registration: NCT01866904.

## 1 INTRODUCTION

Advanced age is a strong determinant of post‐myocardial infarction (MI) mortality in the 6 months after hospital discharge.^1^, ^2^, ^3^ Compared with their younger counterparts, older adults have more comorbid chronic conditions and more functional impairments, both physical and cognitive, which may contribute to this higher mortality risk.^4^, ^5^, ^6^, ^7^ Despite the high prevalence of comorbid chronic conditions and functional impairment in older patients hospitalized for MI, there are relatively limited contemporary data describing the interrelationships between age, multimorbidity, and impairment in function and their association with clinical outcomes. A better understanding of such relationships would not only be useful to stratify patient risk, but also to identify vulnerable populations who may benefit from closer follow‐up, as well as transitional care and multimodality physical function interventions that have been shown to improve outcomes in such patients.^8^ The long‐Term rIsk, clinical manaGement, and healthcare Resource utilization of stable coronary artery dISease in post–myocardial infarction patients (TIGRIS) registry presents an opportunity to determine the prevalence of comorbid chronic conditions and functional impairment among older patients with MI. We specifically sought to evaluate the association between the presence of multimorbidity and functional impairment among older patients with MI, with both cardiovascular (CV) and non‐CV mortality after discharge.

## 2 METHODS

### 2.1 Data source and analysis population

The TIGRIS registry^9^, ^10^ is a prospective, global registry of stable coronary disease of patients aged 50 years or older with a documented history of presumed spontaneous MI 1−3 years before enrollment who have provided written consent and have at least one of the following risk factors: (a) age ≥65 years; (b) diabetes mellitus requiring medication; (c) documented history of a second prior presumed spontaneous MI (>1 year before enrollment); (d) angiographic evidence of multivessel coronary artery disease; and/or (e) non–end‐stage chronic kidney disease (creatinine clearance [using Cockcroft‐Gault equation]: 15−60 ml/min). Patients were excluded if any of the following were present: (a) any condition/circumstance that could significantly limit complete follow‐up of the patient; (b) serious/severe comorbidities that could limit life expectancy (<1 year); (c) ongoing participation in a blinded randomized clinical trial; and/or (d) patients receiving treatment with ticagrelor beyond 12 months post‐MI (which represented off‐label use of ticagrelor at the time of study initiation). The broad goal of the TIGRIS registry was to provide a standardized prospective and longitudinal description of patient characteristics, events (per person‐years at 12 and 24 months), healthcare resource utilization, and current treatment patterns as seen in a relatively unselected patient population after acute coronary syndrome (ACS). The study was performed in accordance with ethical principles consistent with the Declaration of Helsinki, the International Conference on Harmonization Good Clinical Practice Guidelines, and the applicable legislation on nonintervention studies in participating countries. The study protocol and informed consent were reviewed and approved by the corresponding health authorities and ethics boards of all participating study sites. The study was registered at ClinicalTrials.gov (clinical trial identifier NCT01866904).

A standardized electronic case report form was used for data entry. Baseline data included relevant medical history, demographics, details regarding the index MI before enrollment, variables from routine physical examination, and laboratory testing, where available. Patients were contacted every 6 months for a follow‐up period of 2 years either by a phone call or personal visit. All outcome events were confirmed by the treating physician or hospital, including determination of the final diagnosis, primary cause of hospitalization, duration of hospital stay, procedures, and interventions. If a death occurred, efforts were made to identify the cause (CV or non‐CV related) based on the death certificate, if available, or through relatives, physicians, or hospitals. From June 18, 2013 to November 29, 2014, 9225 patients were enrolled by 349 principal investigators in 25 countries from Asia‐Pacific/Australia, Europe, North America, and South America, including 750 patients from China who were excluded from the current analyses following updates to the Human Genetic Resources regulation in China. Only patients ≥65 years of age were included.

### 2.2 Chronic comorbid conditions and assessment of functional impairment

The following comorbid conditions were assessed: congestive heart failure, atrial fibrillation, chronic angina, stroke/transient ischemic attack (TIA), peripheral vascular disease, diabetes mellitus, non−end‐stage renal disease, chronic anemia, gastrointestinal disease (liver disease, gastroesophageal reflux disease, varices), chronic obstructive pulmonary disease, cancer, and depression. Functional impairment was calculated using the five‐dimension EuroQol (EQ‐5D) based on functioning in three domains—mobility, self‐care, and usual activities. Each of these three domains were categorized as no problems (0) and some or severe problems (1). The final functional impairment summary score was a total possible score of 3 (none‐0, mild‐1, moderate‐2, or severe functional impairment‐3).

### 2.3 Statistical analysis

Continuous variables are reported as medians with 25th and 75th percentiles and compared using the Wilcoxon rank‐sum test. Categorical variables are presented as proportions and compared using the *χ* ^2^ test. Baseline characteristics, index‐hospital management, and medical therapy at study enrollment was compared between patients 65−75 and ≥75 years of age, between patients with no, mild, moderate, and severe functional impairment, and between patients with 0−1, 2−3, and ≥4 comorbidities. These data are available as Supporting Information: Tables. Previous TIGRIS publications have demonstrated that clinical outcomes in TIGRIS accumulate steadily over time.^11^, ^12^ Therefore, the association of each of the categories of age, number of comorbid conditions, and degree of functional impairment with the 2‐year incidence of CV outcomes composite that included CV death, MI, stroke, and unstable angina was determined using Poisson regression analysis adjusting for the other two factors, respectively. Equivalent analyses were performed on CV death and non‐CV death. Results are presented as rate ratios (RRs) and their corresponding 95% confidence intervals (CIs). Statistical analyses were performed using Stata version 15.1 (StataCorp).

## 3 RESULTS

Of the 5132 patients aged ≥65 years who were included in the present analysis, the time from index MI to enrollment was 1.8 (interquartile range [IQR] 1.4−2.4) years. The median (IQR) age was 72 (68−76) years; 31.8% (*N* = 5132) of patients were aged ≥75 years. Women represented 27.4% of the population. Most patients were Caucasian (74.4%), 19.4% were Asian, 1.0% were Black, and 5.1% were from other races. Most patients lived in a metropolitan area (63.9%), with 15.9% living alone. Healthcare status (among 99.7% of patients who responded) included government (68.5%), private (14.7%), employer provided (1.7%), a combination of these sources (5.6%), others (4.9%), and none (4.4%).

The prevalence of comorbid conditions was as follows: 29.4% had diabetes, 25.5% had depression, 13.1% had history of heart failure, 9.6% had non–end‐stage renal disease, and 7.4% had stroke/TIA (Table 1). Overall, 29.9% of patients had none of the 12 comorbid conditions and 33.6% had one, 19.5% had two, and 17.0% had ≥3 comorbidities. Self‐reported health status using the EQ‐5D assessment was available for 5108 patients (99.5%), and limitations were identified with mobility in 1501 (29.4%), self‐care in 338 (6.7%), and usual activities in 1039 patients (20.3%). Overall, functional impairment was considered mild in 18.5%, moderate in 10.7%, and severe in 5.5% of patients. Baseline characteristics, index hospital management, and medications at study enrollment by age, number of comorbid conditions, and presence and severity of functional impairment are available in Supporting Information: Tables S1, S2, and S3, respectively.

**Table 1:** Baseline characteristics for patients aged ≥65 years (*N* = 5132)

Characteristic	*n* (%) or mean (SD)
Age (years)	
65−74	3501 (68.2)
75 or older	1631 (31.8%)
Sex	
Male	3727 (72.6%)
Female	1405 (27.4%)
Ethnicity	
Caucasian	3798 (74.4%)
Black	52 (1.0%)
Asian	990 (19.4%)
Other	262 (5.1%)
Region	
Asia and Australia	1237 (24.1%)
Europe	2664 (51.9%)
North America	612 (11.9%)
Latin America	619 (12.1%)
BMI (kg/m^2^)	27.1 (4.5)
Waist circumference (cm)	98.3 (12.8)
SBP (mmHg)	133.0 (18.2)
DBP (mmHg)	75.6 (10.6)
Smoking status	
Never smoked	2094 (40.8%)
Former smoker	2542 (49.5%)
Current smoker	496 (9.7%)
Heart rate (bpm)	67.3 (10.8)
Diabetes requiring medication	1339 (26.1%)
Functional impairment	
EQ‐5D mobility	
No problems	3608 (70.6%)
Some or severe problems	1501 (29.4%)
EQ‐5D self‐care	
No problems	4771 (93.4%)
Some or severe problems	338 (6.6%)
EQ‐5D usual activities	
No problems	4069 (79.7%)
Some or severe problems	1039 (20.3%)
Functional impairment summary score (0−3)^a^	
None	3338 (65.3%)
Mild	943 (18.5%)
Moderate	547 (10.7%)
Severe	280 (5.5%)
Comorbid conditions	
CHF	672 (13.1%)
Atrial fibrillation	569 (11.1%)
Chronic angina	511 (10.0%)
Stroke or TIA	382 (7.4%)
Of which stroke	262 (5.1%)
Of which TIA	142 (2.8%)
PVD	390 (7.6%)
Diabetes	1508 (29.4%)
Non–end‐stage renal disease	495 (9.6%)
Chronic anemia	186 (3.6%)
GI conditions (liver disease, peptic ulcer disease, esophageal varices)	212 (4.1%)
COPD	434 (8.5%)
Cancer	487 (9.5%)
Depression	1303 (25.5%)
EQ‐5D anxious	1116 (21.8%)
Antidepressant use	369 (7.2%)
Number of comorbid conditions	
0	1535 (29.9%)
1	1725 (33.6%)
2	1001 (19.5%)
3	504 (9.8%)
4	225 (4.4%)
5	87 (1.7%)
6	33 (0.6%)
7	12 (0.2%)
8	9 (0.2%)
9	1 (0.0%)

Abbreviations: BMI, body mass index; bpm, beats per minute; CHF, congestive heart failure; COPD, chronic obstructive pulmonary disease; DBP, diastolic blood pressure; EQ‐5D, five‐dimension EuroQol; GI, gastrointestinal; PVD, peripheral vascular disease; SBP, systolic blood pressure; TIA, transient ischemic attack.

^a^ Created by adding each of the three EQ‐5D components (mobility, self‐care, and usual activities) scored as 0 (no problems) or 1 (some or severe problems).

### 3.1 Association between older age, multimorbidity, and functional impairment

**Figure 1:**
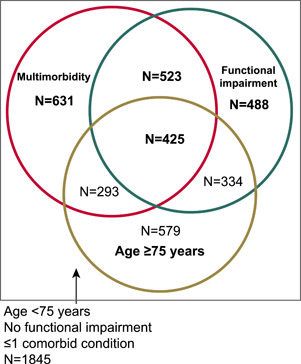
Overlap of age, multimorbidity, and functional impairment. Functional impairment: at least some problem in one of the three relevant EQ‐5D components. EQ‐5D, 5‐dimension EuroQol.

There was a significant overlap in the domains of age, number of comorbidities, and functional impairment (Figure 1). Patients older than 75 years of age had a greater number of comorbidities per patient compared with those aged 65−74 years (mean 1.6 vs. 1.2) (Supporing Information: Table S4A). The degree of functional impairment was greater with a greater number of comorbidities (Supporing Information: Table S4B). Patients older than 75 years of age had greater functional impairment compared with those aged 65−74 years (mean functional impairment score 0.8 vs. 0.5) (Supporing Information: Table S4C).

### 3.2 Association of age, multimorbidity, and functional impairment with clinical outcomes

**Figure 2:**
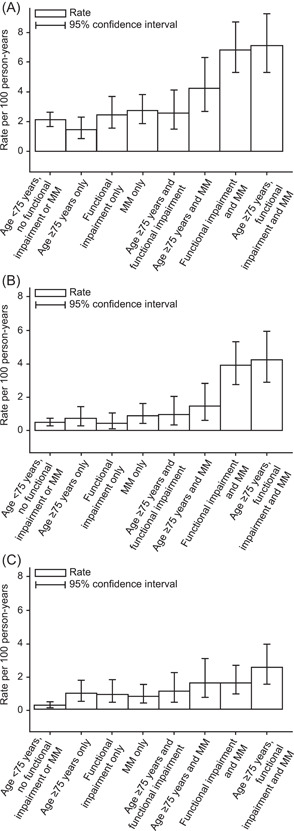
Rate of events by age, functional impairment, and MM. (A) CV composite outcome (CV death, MI, stroke, or unstable angina). (B) CV death. (C) Non‐CV death. CV, cardiovascular; MI, myocardial infarction; MM, multimorbidity.

**Figure 3:**
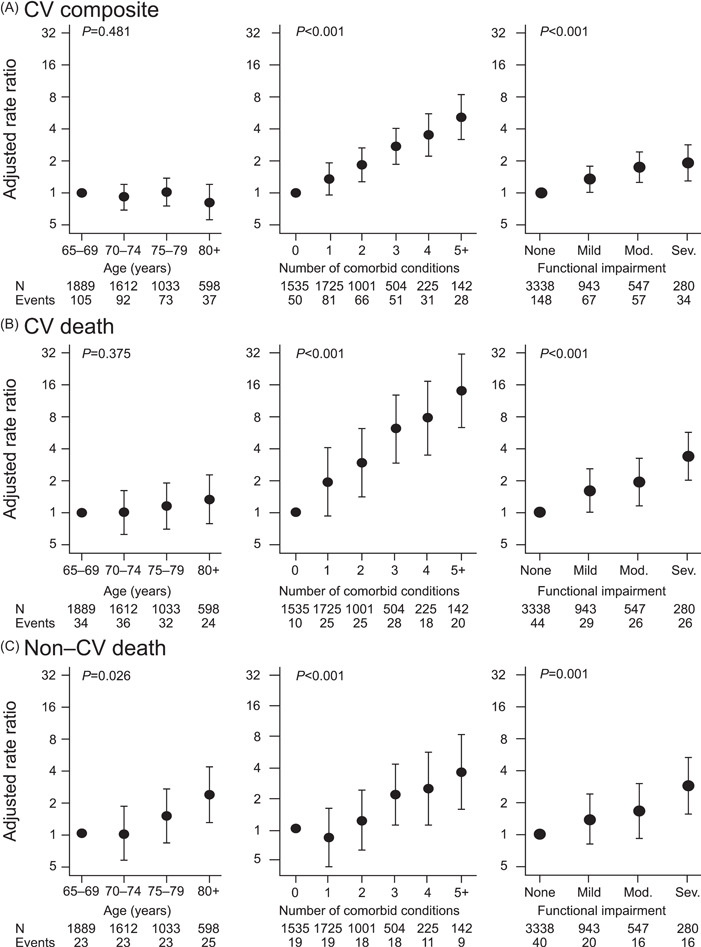
Adjusted* rate ratios for age, multimorbidity, and functional impairment. (A) CV composite (CV death, MI, stroke, urgent revascularization). (B) CV death. (C) Non‐CV death. *Age, multimorbidity, and functional impairment were mutually adjusted for the other variables. CV, cardiovascular; MI, myocardial infarction; Mod., moderate; Sev., severe.

Crude rates of the CV composite outcome were lowest for patients without comorbidities or functional impairment, irrespective of age. Similarly, the rates of the CV composite outcome were greatest for patients with multimorbidity and functional impairment, irrespective of age (Figure 2). The rate of the CV composite outcome was 3.6 per 100 patient‐years in patients over 75 years of age compared with 3.0 in patients aged 65−74 years; this difference was not statistically significant after adjusting for higher levels of functional impairment and greater number of comorbidities (adjusted RR 0.99, 95% CI: 0.78−1.24) (Figure 3). Adjusted rates of non‐CV but not CV mortality increased with increasing age (Figure 3).

Greater functional impairment was associated with a higher rate of the CV composite outcome (Figure  3). Greater functional impairment was also associated with a higher and relatively equal magnitude risk of CV and non‐CV mortality (Figure 3). Adjusted RRs per one‐step increase in functional impairment were 1.28 (95% CI: 1.14−1.43) for the CV composite, 1.52 (95% CI: 1.29−1.79) for CV death, and 1.42 (95% CI: 1.17−1.73) for non‐CV death. Rates of the CV composite outcome increased with increasing number of comorbidities (Figure 3). The association between number of comorbid conditions and mortality was stronger for CV mortality compared with non‐CV mortality (Figure 3,3). Adjusted RRs per additional comorbidity were 1.33 (95% CI: 1.25−1.42) for the CV composite, 1.52 (95% CI: 1.38−1.67) for CV death, and 1.29 (1.14−1.47) for non‐CV death.

## 4 DISCUSSION

The data from the international TIGRIS registry provide a unique perspective on the prevalence of multimorbidity and functional impairment in older patients 1−3 years post‐MI and their association with clinical outcomes including mortality. Unsurprisingly, older age is associated with a greater number of comorbidities and functional impairment; however, after adjustment for these comorbidities and functional impairment, increasing age was associated with non‐CV but not CV mortality. On the contrary, the number of comorbidities was more strongly associated with CV than non‐CV mortality. The functional impairment was equally associated with CV and non‐CV mortality. These novel findings highlight the importance of screening patients not only for their comorbidities but also for functional impairment and suggest the need for tailored management of chronic conditions and functional impairment to optimize both CV and non‐CV outcomes in older patients post‐MI. Our findings are echoed by a recent statement from the American Heart Association suggesting that a thoughtful approach to critical care management in patients with CV disease requires consideration of frailty and multimorbidity.^13^

Age is a well‐recognized predictor of mortality, both in‐hospital and after discharge.^14^, ^15^, ^16^ However, risk‐prediction models of post‐MI patients focus on the acute phase after the index event, with limited data available beyond the first year. In addition, there is little, if any, inclusion of data on functional impairment, cognition, and frailty in these risk‐prediction models, which include factors now well recognized to be prevalent in the older and associated with worse outcomes.^7^, ^17^ Furthermore, prior risk scores have traditionally not differentiated between CV versus non‐CV mortality risks. In this study of patients 1−3 years post‐MI, we found that after adjustment for comorbidities and functional impairment, increasing age by itself was not associated with increased CV mortality. The association of age in this population of patients >65 years of age was much stronger with non‐CV mortality. Most importantly, mortality rates were low among patients without comorbidities or functional impairment, irrespective of age.

In the ComprehenSIVe Evaluation of Risk Factors in Older Patients with Acute Myocardial Infarction (SILVER‐AMI) study, functional mobility and hearing impairment were independently associated with mortality at 6 months after discharge among patients with MI aged ≥75 years.^6^ The association between hearing impairment and mortality may be mediated by patients' difficulty in comprehending complex medications and follow‐up instructions at the time of hospital discharge. Mobility is a key measure of frailty. Frailty has been associated with worse clinical outcomes after discharge from the hospital post‐MI.^17^ In a 10‐year longitudinal, nationally representative study of hospital admissions among seniors having Medicare, approximately half had functional impairments, which are associated with higher readmission rates, especially among patients admitted for heart failure, MI, or pneumonia.^18^ This unmeasured functional impairment may play a key mechanistic role in what has been described as post‐hospitalization syndrome, which is a condition of elevated generalized risk for poor health outcomes within 30 days of discharge due to the patients' inability to care for themselves, manage their affairs, and recover from their hospitalization that leads to readmission shortly after discharge.^19^ In our study cohort, functional impairment was present in 33.4% of patients, with impairment of mobility in 29.4%. Despite differences in the definition of functional impairment between studies, consistent with prior findings, the presence and degree of functional impairment was associated with greater CV and non‐CV mortality in our study cohort. The strength of association with CV and non‐CV mortality was similar. In addition to higher risk of mortality, functional mobility is also a strong predictor of 30‐day readmission.^20^ Functional impairment on admission is mostly overlooked but is a highly suitable target for interventions to reduce readmission and mortality. Transitional care and multimodality physical function interventions have been shown to improve outcomes in older patients with these disorders.^8^ Further research is needed to determine if therapeutic interventions and changes in healthcare delivery designed to alter the course of functional impairment would improve survival.

Despite the high prevalence of medical comorbidities in patients hospitalized for ACS, there are relatively limited contemporary data describing the possible age‐specific differences in the effects of cardiac‐ and noncardiac‐related conditions on the risk of CV and non‐CV deaths. In the Transitions, Risks, and Actions in Coronary Events Center for Outcomes Research and Education (TRACE‐CORE) study of patients hospitalized with ACS, approximately two of five patients who were discharged had four or more diagnosed morbidities. Patients presenting with multiple chronic conditions were more likely to report stress, depression, and anxiety; these patients with multiple morbidities had a lower quality of life, had low health literacy and numeracy, and were more likely to be cognitively impaired than patients with no or just one morbidity.^21^ Patients with multiple morbidities have been shown to be at substantial risk of disability, death, and poor quality of life and account for a disproportionate share of health expenditure in the United States.^4^, ^22^ In the current study cohort, only 16.3% of patients had ≥3 comorbidities. This is likely due to the selection of patients for this registry, where patients with serious/severe comorbidities that could limit life expectancy (<1 year) or follow‐up were excluded from participation. Despite the lower prevalence of comorbidities in the current population compared with other cohorts having post‐MI patients of a similar age, we observed a strong association between the increasing number of comorbidities and higher mortality, with a stronger association for CV than non‐CV mortality.

The clinical management of patients with MI having multiple medical morbidities and/or functional impairment is particularly challenging, partly due to a high risk for adverse events as well as the need for complex and individualized therapeutic regimens. In a study of nearly 3000 patients with AMI treated at 53 hospitals in Ontario, Canada, between 1999 and 2003, the number of preexisting conditions was inversely associated with the receipt of coronary reperfusion therapy.^23^ A possible explanation for this treatment risk paradox may have been due to physicians being generally averse to providing more aggressive treatments to patients with multiple comorbidities because they perceive these interventions as risky or futile due to the complexity as well as the overall fragile conditions of patients. However, given the increased risk of CV mortality with the increasing number of comorbidities, even after adjustment for age and functional impairment, it is imperative that additional attention is paid to patients with multiple comorbidities so that they may benefit from more intensive follow‐up and risk‐factor modifications and have better outcomes.

### 4.1 Limitations

First, enrollment in this registry was nonconsecutive; thus, older patients with greater comorbidities or functional impairment may be less likely to be enrolled. In addition, the study excluded patients with comorbidities limiting complete follow‐up or survival to 1 year. Second, only a select group of comorbidities were measured; thus, the associations with mortality may be affected by additional unmeasured comorbidities not accounted for in this study cohort. Third, classification of functional impairment was taken from a chart review and not confirmed by formal testing. Fourth, study results were also not adjusted for other potentially confounding variables including socioeconomic status, medical/revascularization therapy, medication use, health literacy, and other factors associated with long‐term health and outcomes. Fifth, the effect of multiple comorbidities and functional impairment on the occurrence of the index MI cannot be determined from this study. Prior studies suggest a complicated etiological relationship among development of ACS, multimorbidity, and psychosocial illnesses. Many chronic diseases that are independent risk factors for an MI may also cause stress, depression, anxiety, and functional decline. Alternatively, the presence of psychosocial difficulties may also precipitate the onset of ACS. Sixth, patients 1−3 years post‐MI were included in this study cohort; thus, findings do not consider patients who died within 1 year post‐MI. Early death post‐MI is more likely to be a CV than a non‐CV cause; thus, in this population of MI patients at least 1 year post‐MI, the associations observed with CV mortality are likely less strong than would have been in a cohort of MI patients after discharge from their index presentation.

## 5 CONCLUSIONS

Multimorbidity and functional impairments are common among older patients post‐MI. After adjustment for comorbidities and functional impairment, older age was associated with non‐CV mortality The association of the number of comorbidities was much stronger with CV mortality than with non‐CV mortality while functional impairment was equally associated with CV and non‐CV mortality. These findings highlight the importance of considering comorbid conditions and functional impairment as predictors of risk for adverse outcomes and aspects of medical decision making.

## AUTHOR CONTRIBUTIONS

**Akshay Bagai**: conceptualization, data curation, formal analysis, funding acquisition, project administration, writing (original draft), writing (review), and editing. **Faeez M. Ali**: conceptualization, data curation, writing (original draft), writing (review), and editing. **John Gregson**: conceptualization, data curation, formal analysis, funding acquisition, methodology, project administration, validation, writing (original draft), writing (review), and editing. **Karen P. Alexander**: conceptualization, writing (review), and editing. **Mauricio G. Cohen**: data curation, funding acquisition, project administration, writing (review), and editing. **Karolina Andersson Sundell**: writing (review) and editing. **Tabassome Simon**: data curation, funding acquisition, project administration, writing (review), and editing. Dirk Westermann: data curation, funding acquisition, project administration, writing (review), and editing. **Satoshi Yasuda**: data curation, funding acquisition, project administration, writing (review), and editing. **David Brieger**: data curation, funding acquisition, project administration, writing (review), and editing. **Shaun G. Goodman**: data curation, funding acquisition, project administration, writing (review), and editing. **Jose C. Nicolau**: data curation, funding acquisition, project administration, writing (review), and editing. **Christopher B. Granger**: data curation, funding acquisition, project administration, writing (review), and editing. **Stuart Pocock**: conceptualization, data curation, formal analysis, funding acquisition, methodology, project administration, supervision, validation, writing (original draft), writing (review), and editing.

## CONFLICTS OF INTEREST

A. B. has received speaker/consulting honoraria from AstraZeneca, Bayer Inc, Servier, Bristol Myers Squibb/Eli Lilly, Boehringer Ingelheim, HLS Therapeutics, and JAMP Pharma. J. G. received research funding from AstraZeneca for the work under consideration. M. G. C. has received speaker/consulting honoraria and/or research grant support from AstraZeneca, Medtronic, Abiomed, and Merit Medical. K. A. S. is an employee of AstraZeneca. T. S. has received speaker/consulting honoraria and/or research grant support from Astellas, Amgen, AstraZeneca, Bayer, Boehringer Ingelheim, Eli Lilly, GlaxoSmithKline, Merck, Novartis, Pfizer, and Sanofi. D. W. has received speaker/consulting honoraria and/or research grant support from AstraZeneca, Bayer, Berlin‐Chemie, Biotronik, and Novartis. S. Y. has received speaker/consulting honoraria and/or research grant support from Takeda, Daiichi Sankyo, AstraZeneca, Boehringer Ingelheim, and BMS. D. B. has received speaker/consulting honoraria and/or research grant support from Amgen, AstraZeneca, Bayer, Boehringer Ingelheim, BMS, Eli Lilly, Merck, and Sanofi. S. G. G. has received research grant support (e.g., steering committee or data and safety monitoring committee) and/or speaker/consulting honoraria (e.g., advisory boards) from: Amgen, Anthos Therapeutics, AstraZeneca, Bayer, Boehringer Ingelheim, Bristol Myers Squibb, CSL Behring, Daiichi‐Sankyo/American Regent, Eli Lilly, Esperion, Ferring Pharmaceuticals, HLS Therapeutics, JAMP Pharma, Merck, Novartis, Novo Nordisk A/C, Pendopharm/Pharmascience, Pfizer, Regeneron, Sanofi, Servier, Valeo Pharma; and salary support/honoraria from the Heart and Stroke Foundation of Ontario/University of Toronto (Polo) Chair, Canadian Heart Research Centre and MD Primer, Canadian VIGOUR Centre, Cleveland Clinic Coordinating Centre for Clinical Research, Duke Clinical Research Institute, New York University Clinical Coordinating Centre, PERFUSE Research Institute, TIMI Study Group (Brigham Health). J. C. N. has received speaker/consulting honoraria and/or research grant support from Amgen, AstraZeneca, Bayer, Esperion, CLS Behring, Dalcor, Daiichi Sankyo, Janssen, Novartis, NovoNordisk, Sanofi, Servier, and Vifor. C. B. G. has received consulting honoraria and/or research grant support from Armetheon, AstraZeneca, Bayer, Boehringer Ingelheim, BMS, Daiichi Sankyo, Eli Lilly, Gilead, GlaxoSmithKline, Hoffmann‐La Roche, Janssen, Medtronic, Pfizer, Salix Pharmaceuticals, Sanofi, Takeda, and The Medicines Company. S. P. has received research grant support from AstraZeneca. The remaining authors declare no conflict of interest.

## Supporting information

Supporting information.

## Data Availability

The data that support the findings of this study are available from the authors upon reasonable request. Baseline patient characteristics and data on long‐term oral antiplatelet use and event rates in the TIGRIS registry have been published elsewhere (DOIs: 10.1002/clc.22837, 10.1016/j.ijcard.2017.02.062, and 10.1002/clc.23283).
