# Supplementary material for: Multimorbidity, functional impairment, and mortality in older patients stable after prior acute myocardial infarction: Insights from the TIGRIS registry
Source: Clin Cardiol. 2022 Nov 1;45(12):1277–86. doi: 10.1002/clc.23915 (PMC9748748; doi:10.1002/clc.23915)
Supplement: Supplementary file 1 — Supporting information. [file CLC-45-1277-s001.docx]

**Supplementary material**

**Multimorbidity, functional impairment, and mortality in older patients stable after prior acute myocardial infarction: Insights from the TIGRIS registry**

**Running title:** Multimorbidity and functional impairment in older patients with MI

Akshay Bagai, MD^1*^, Faeez Mohamad Ali, MD^1^, John Gregson, PhD^2^, Karen P. Alexander, MD^3^, Mauricio G. Cohen, MD^4^, Karolina Andersson Sundell, PhD^5^, Tabassome Simon, MD, PhD^6, 7^, Dirk Westermann, MD^8^, Satoshi Yasuda, MD^9^, David Brieger, MBBS, PhD^10^, Shaun G. Goodman, MD, MSc^1,11^, Jose C. Nicolau, MD, PhD^12^, Christopher B. Granger, MD^13^, Stuart Pocock, PhD^2^

**Table S1.** Baseline characteristics by age for patients aged ≥65 years (N=5132)

| **Characteristic** | **n/N (%)** | | **p-value** |
| --- | --- | --- | --- |
|  | **Age 65–74 years** | **Age 75+ years** |  |
| N | 3501 | 1631 |  |
| Age | 69.3 (2.8) | 79.1 (3.6) | <0.0001 |
| Sex |  |  | <0.0001 |
| Male | 2633/3501 (75.2) | 1094/1631 (67.1) |  |
| Female | 868/3501 (24.8) | 537/1631 (32.9) |  |
| Ethnicity |  |  | 0.0007 |
| Caucasian | 2650/3482 (76.1) | 1148/1620 (70.9) |  |
| Black | 36/3482 (1.0) | 16/1620 (1.0) |  |
| Asian/Oriental | 625/3482 (17.9) | 365/1620 (22.5) |  |
| Other | 171/3482 (4.9) | 91/1620 (5.6) |  |
| Region |  |  | 0.0060 |
| Asia and Australia | 808/3501 (23.1) | 429/1631 (26.3) |  |
| Europe | 1875/3501 (53.6) | 789/1631 (48.4) |  |
| North America | 404/3501 (11.5) | 208/1631 (12.8) |  |
| Latin America | 414/3501 (11.8) | 205/1631 (12.6) |  |
| BMI (kg/m^2^) | 27.4 (4.6) | 26.5 (4.4) | <0.0001 |
| Waist circumference (cm) | 98.7 (12.8) | 97.3 (12.9) | 0.0009 |
| SBP (mmHg) | 133.0 (18.0) | 133.1 (18.7) | 0.93 |
| DBP (mmHg) | 76.5 (10.4) | 73.7 (10.6) | <0.0001 |
| Smoking status |  |  | <0.0001 |
| Never smoked | 1322/3501 (37.8) | 772/1631 (47.3) |  |
| Former smoker | 1768/3501 (50.5) | 774/1631 (47.5) |  |
| Current smoker | 411/3501 (11.7) | 85/1631 (5.2) |  |
| Heart rate (bpm) | 67.3 (10.8) | 67.4 (10.9) | 0.62 |
| Diabetes requiring medication | 919/3501 (26.2) | 420/1631 (25.8) | 0.70 |
| Second prior MI | 333/3501 (9.5) | 180/1631 (11.0) | 0.090 |
| Multiple-vessel disease | 1919/3501 (54.8) | 929/1631 (57.0) | 0.15 |
| CKD | 266/3501 (7.6) | 229/1631 (14.0) | <0.0001 |
| Type of MI |  |  | 0.0004 |
| STEMI | 1777/3501 (50.8) | 731/1631 (44.8) |  |
| NSTEMI | 1542/3501 (44.0) | 810/1631 (49.7) |  |
| Unknown | 182/3501 (5.2) | 90/1631 (5.5) |  |
| MI management |  |  | 0.0002 |
| PCI | 2811/3501 (80.3) | 1246/1631 (76.4) |  |
| CABG | 274/3501 (7.8) | 122/1631 (7.5) |  |
| Medical | 416/3501 (11.9) | 263/1631 (16.1) |  |
| Hyperlipidemia | 2449/3501 (70.0) | 1146/1631 (70.3) | 0.82 |
| Hypertension | 2566/3501 (73.3) | 1303/1631 (79.9) | <0.0001 |
| CABG | 535/3501 (15.3) | 280/1631 (17.2) | 0.085 |
| PCI | 2998/3501 (85.6) | 1329/1631 (81.5) | 0.0001 |
| Venous thrombo-embolism | 63/3501 (1.8) | 44/1631 (2.7) | 0.036 |
| Atrial fibrillation | 338/3501 (9.7) | 231/1631 (14.2) | <0.0001 |
| PVD | 229/3501 (6.5) | 161/1631 (9.9) | <0.0001 |
| COPD | 287/3501 (8.2) | 147/1631 (9.0) | 0.33 |
| ACE inhibitor/ARB | 2675/3501 (76.4) | 1219/1631 (74.7) | 0.19 |
| Antiplatelet medication |  |  | 0.14 |
| No APT | 217/3501 (6.2) | 125/1631 (7.7) |  |
| SAPT | 2510/3501 (71.7) | 1158/1631 (71.0) |  |
| DAPT | 774/3501 (22.1) | 348/1631 (21.3) |  |
| Anticoagulant | 224/3501 (6.4) | 162/1631 (9.9) | <0.0001 |
| Beta-blocker | 2794/3501 (79.8) | 1284/1631 (78.7) | 0.37 |
| Diuretic | 883/3501 (25.2) | 581/1631 (35.6) | <0.0001 |
| Statin | 3214/3501 (91.8) | 1481/1631 (90.8) | 0.23 |

Data are presented as n/N (%) unless otherwise specified.

ACE, angiotensin-converting enzyme; APT, antiplatelet therapy; ARB, angiotensin receptor blocker; BMI, body mass index; bpm, beats per minute; CABG, coronary artery bypass graft; CHF, congestive heart failure; CKD, chronic kidney disease; COPD, chronic obstructive pulmonary disease; DAPT, dual antiplatelet therapy; DBP, diastolic blood pressure; MI, myocardial infarction; NSTEMI, non-ST-segment elevation MI; PCI, percutaneous coronary intervention; PVD, peripheral vascular disease; SAPT, single antiplatelet therapy; SBP, systolic blood pressure; STEMI, ST-segment elevation MI.

**Table S2.** Baseline characteristics by functional impairment score

| **Characteristic** | **Number of components with impairment** | | | | **p-value** |
| --- | --- | --- | --- | --- | --- |
|  | **0 components** | **1 component** | **2 components** | **3 components** |  |
| N | 3338 | 943 | 547 | 280 |  |
| Age | 71.6 (5.1) | 73.6 (5.7) | 73.9 (5.9) | 75.2 (6.4) | <0.0001 |
| Sex |  |  |  |  | <0.0001 |
| Male | 2592/3338 (77.7) | 634/943 (67.2) | 327/547 (59.8) | 155/280 (55.4) |  |
| Female | 746/3338 (22.3) | 309/943 (32.8) | 220/547 (40.2) | 125/280 (44.6) |  |
| Ethnicity |  |  |  |  | 0.11 |
| Caucasian | 2465/3314 (74.4) | 695/939 (74.0) | 415/545 (76.1) | 204/280 (72.9) |  |
| Black | 34/3314 (1.0) | 9/939 (1.0) | 8/545 (1.5) | 1/280 (0.4) |  |
| Asian/Oriental | 665/3314 (20.1) | 172/939 (18.3) | 92/545 (16.9) | 56/280 (20.0) |  |
| Other | 150/3314 (4.5) | 63/939 (6.7) | 30/545 (5.5) | 19/280 (6.8) |  |
| Region |  |  |  |  | 0.096 |
| Asia and Australia | 828/3338 (24.8) | 212/943 (22.5) | 127/547 (23.2) | 64/280 (22.9) |  |
| Europe | 1736/3338 (52.0) | 494/943 (52.4) | 264/547 (48.3) | 155/280 (55.4) |  |
| North America | 395/3338 (11.8) | 114/943 (12.1) | 79/547 (14.4) | 23/280 (8.2) |  |
| Latin America | 379/3338 (11.4) | 123/943 (13.0) | 77/547 (14.1) | 38/280 (13.6) |  |
| BMI (kg/m^2^) | 26.7 (4.1) | 27.6 (4.8) | 28.5 (5.5) | 28.1 (5.3) | <0.0001 |
| Waist circumference (cm) | 96.9 (12.1) | 100.2 (13.6) | 102.4 (13.7) | 100.3 (14.2) | <0.0001 |
| SBP (mmHg) | 133.0 (17.9) | 133.5 (19.0) | 133.2 (18.6) | 131.9 (18.9) | 0.63 |
| DBP (mmHg) | 75.9 (10.4) | 75.2 (10.8) | 74.8 (10.8) | 74.6 (11.4) | 0.018 |
| Smoking status |  |  |  |  | 0.021 |
| Never Smoked | 1362/3338 (40.8) | 363/943 (38.5) | 218/547 (39.9) | 139/280 (49.6) |  |
| Former Smoker | 1662/3338 (49.8) | 490/943 (52.0) | 266/547 (48.6) | 113/280 (40.4) |  |
| Current Smoker | 314/3338 (9.4) | 90/943 (9.5) | 63/547 (11.5) | 28/280 (10.0) |  |
| Heart rate (bpm) | 66.4 (10.5) | 68.6 (11.1) | 69.6 (11.5) | 69.4 (11.3) | <0.0001 |
| Diabetes requiring medication | 759/3338 (22.7) | 298/943 (31.6) | 172/547 (31.4) | 103/280 (36.8) | <0.0001 |
| Second prior MI | 298/3338 (8.9) | 106/943 (11.2) | 74/547 (13.5) | 35/280 (12.5) | 0.0016 |
| Multiple-vessel disease | 1802/3338 (54.0) | 548/943 (58.1) | 310/547 (56.7) | 173/280 (61.8) | 0.015 |
| CKD | 254/3338 (7.6) | 103/943 (10.9) | 84/547 (15.4) | 52/280 (18.6) | <0.0001 |
| Type of MI |  |  |  |  | <0.0001 |
| STEMI | 1710/3338 (51.2) | 430/943 (45.6) | 233/547 (42.6) | 122/280 (43.6) |  |
| NSTEMI | 1441/3338 (43.2) | 471/943 (49.9) | 288/547 (52.7) | 143/280 (51.1) |  |
| Unknown | 187/3338 (5.6) | 42/943 (4.5) | 26/547 (4.8) | 15/280 (5.4) |  |
| MI management |  |  |  |  | <0.0001 |
| PCI | 2724/3338 (81.6) | 718/943 (76.1) | 385/547 (70.4) | 207/280 (73.9) |  |
| CABG | 255/3338 (7.6) | 74/943 (7.8) | 47/547 (8.6) | 20/280 (7.1) |  |
| Medical | 359/3338 (10.8) | 151/943 (16.0) | 115/547 (21.0) | 53/280 (18.9) |  |
| Hyperlipidemia | 2347/3338 (70.3) | 660/943 (70.0) | 392/547 (71.7) | 183/280 (65.4) | 0.29 |
| Hypertension | 2446/3338 (73.3) | 736/943 (78.0) | 437/547 (79.9) | 236/280 (84.3) | <0.0001 |
| CABG | 499/3338 (14.9) | 159/943 (16.9) | 114/547 (20.8) | 43/280 (15.4) | 0.0048 |
| PCI | 2872/3338 (86.0) | 783/943 (83.0) | 427/547 (78.1) | 222/280 (79.3) | <0.0001 |
| Venous thrombo-embolism | 53/3338 (1.6) | 27/943 (2.9) | 18/547 (3.3) | 8/280 (2.9) | 0.0089 |
| Atrial fibrillation | 300/3338 (9.0) | 118/943 (12.5) | 95/547 (17.4) | 55/280 (19.6) | <0.0001 |
| PVD | 162/3338 (4.9) | 103/943 (10.9) | 88/547 (16.1) | 35/280 (12.5) | <0.0001 |
| COPD | 211/3338 (6.3) | 111/943 (11.8) | 72/547 (13.2) | 39/280 (13.9) | <0.0001 |
| ACE inhibitor/ARB | 2549/3338 (76.4) | 712/943 (75.5) | 406/547 (74.2) | 211/280 (75.4) | 0.72 |
| Antiplatelet medication |  |  |  |  | <0.0001 |
| No APT | 190/3338 (5.7) | 69/943 (7.3) | 46/547 (8.4) | 36/280 (12.9) |  |
| SAPT | 2438/3338 (73.0) | 661/943 (70.1) | 371/547 (67.8) | 182/280 (65.0) |  |
| DAPT | 710/3338 (21.3) | 213/943 (22.6) | 130/547 (23.8) | 62/280 (22.1) |  |
| Anticoagulant | 214/3338 (6.4) | 77/943 (8.2) | 55/547 (10.1) | 38/280 (13.6) | <0.0001 |
| Beta-blocker | 2649/3338 (79.4) | 759/943 (80.5) | 434/547 (79.3) | 220/280 (78.6) | 0.86 |
| Diuretic | 777/3338 (23.3) | 325/943 (34.5) | 224/547 (41.0) | 136/280 (48.6) | <0.0001 |
| Statin | 3091/3338 (92.6) | 846/943 (89.7) | 495/547 (90.5) | 243/280 (86.8) | 0.0005 |

Data are presented as n/N (%) unless otherwise specified. ACE, angiotensin-converting enzyme; APT, antiplatelet therapy; ARB, angiotensin receptor blocker; BMI, body mass index; bpm, beats per minute; CABG, coronary artery bypass graft; CHF, congestive heart failure; CKD, chronic kidney disease; COPD, chronic obstructive pulmonary disease; DAPT, dual antiplatelet therapy; DBP, diastolic blood pressure; MI, myocardial infarction; NSTEMI, non-ST-segment elevation MI; PCI, percutaneous coronary intervention; PVD, peripheral vascular disease; SAPT, single antiplatelet therapy; SBP, systolic blood pressure; STEMI, ST-segment elevation MI.

**Table S3.** Baseline characteristics by number of comorbid conditions

| **Characteristic** | **Number of comorbid conditions** | | | **p-value** |
| --- | --- | --- | --- | --- |
|  | **0–1 condition** | **2–3 conditions** | **4+ conditions** |  |
| N | 1535 | 2726 | 871 |  |
| Age | 71.6 (5.2) | 72.4 (5.5) | 73.9 (5.8) | <0.0001 |
| Sex |  |  |  | <0.0001 |
| Male | 1197/1535 (78.0) | 1922/2726 (70.5) | 608/871 (69.8) |  |
| Female | 338/1535 (22.0) | 804/2726 (29.5) | 263/871 (30.2) |  |
| Ethnicity |  |  |  | 0.0007 |
| Caucasian | 1163/1527 (76.2) | 1989/2711 (73.4) | 646/864 (74.8) |  |
| Black | 11/1527 (0.7) | 25/2711 (0.9) | 16/864 (1.9) |  |
| Asian/Oriental | 276/1527 (18.1) | 571/2711 (21.1) | 143/864 (16.6) |  |
| Other | 77/1527 (5.0) | 126/2711 (4.6) | 59/864 (6.8) |  |
| Region |  |  |  | 0.0003 |
| Asia and Australia | 346/1535 (22.5) | 710/2726 (26.0) | 181/871 (20.8) |  |
| Europe | 838/1535 (54.6) | 1371/2726 (50.3) | 455/871 (52.2) |  |
| North America | 187/1535 (12.2) | 297/2726 (10.9) | 128/871 (14.7) |  |
| Latin America | 164/1535 (10.7) | 348/2726 (12.8) | 107/871 (12.3) |  |
| BMI (kg/m^2^) | 26.7 (4.2) | 27.2 (4.5) | 27.8 (5.1) | <0.0001 |
| Waist circumference (cm) | 97.0 (12.2) | 98.4 (12.6) | 100.4 (14.2) | <0.0001 |
| SBP (mmHg) | 132.8 (17.9) | 133.4 (18.2) | 132.3 (19.0) | 0.28 |
| DBP (mmHg) | 76.3 (10.1) | 75.8 (10.5) | 74.1 (11.3) | <0.0001 |
| Smoking status |  |  |  | 0.077 |
| Never smoked | 631/1535 (41.1) | 1139/2726 (41.8) | 324/871 (37.2) |  |
| Former smoker | 771/1535 (50.2) | 1314/2726 (48.2) | 457/871 (52.5) |  |
| Current smoker | 133/1535 (8.7) | 273/2726 (10.0) | 90/871 (10.3) |  |
| Heart rate (bpm) | 65.8 (10.4) | 67.6 (10.8) | 69.2 (11.4) | <0.0001 |
| Diabetes requiring medication | 0/1535 (0.0) | 860/2726 (31.5) | 479/871 (55.0) | <0.0001 |
| Second prior MI | 93/1535 (6.1) | 272/2726 (10.0) | 148/871 (17.0) | <0.0001 |
| Multiple-vessel disease | 778/1535 (50.7) | 1510/2726 (55.4) | 560/871 (64.3) | <0.0001 |
| CKD | 0/1535 (0.0) | 211/2726 (7.7) | 284/871 (32.6) | <0.0001 |
| Type of MI |  |  |  | <0.0001 |
| STEMI | 798/1535 (52.0) | 1349/2726 (49.5) | 361/871 (41.4) |  |
| NSTEMI | 665/1535 (43.3) | 1224/2726 (44.9) | 463/871 (53.2) |  |
| Unknown | 72/1535 (4.7) | 153/2726 (5.6) | 47/871 (5.4) |  |
| MI management |  |  |  | <0.0001 |
| PCI | 1303/1535 (84.9) | 2151/2726 (78.9) | 603/871 (69.2) |  |
| CABG | 107/1535 (7.0) | 211/2726 (7.7) | 78/871 (9.0) |  |
| Medical | 125/1535 (8.1) | 364/2726 (13.4) | 190/871 (21.8) |  |
| Hyperlipidemia | 1000/1535 (65.1) | 1934/2726 (70.9) | 661/871 (75.9) | <0.0001 |
| Hypertension | 1040/1535 (67.8) | 2078/2726 (76.2) | 751/871 (86.2) | <0.0001 |
| CABG | 194/1535 (12.6) | 419/2726 (15.4) | 202/871 (23.2) | <0.0001 |
| PCI | 1345/1535 (87.6) | 2298/2726 (84.3) | 684/871 (78.5) | <0.0001 |
| Venous thrombo-embolism | 20/1535 (1.3) | 56/2726 (2.1) | 31/871 (3.6) | 0.0010 |
| Atrial fibrillation | 0/1535 (0.0) | 273/2726 (10.0) | 296/871 (34.0) | <0.0001 |
| PVD | 0/1535 (0.0) | 153/2726 (5.6) | 237/871 (27.2) | <0.0001 |
| COPD | 0/1535 (0.0) | 202/2726 (7.4) | 232/871 (26.6) | <0.0001 |
| ACE inhibitor/ARB | 1123/1535 (73.2) | 2100/2726 (77.0) | 671/871 (77.0) | 0.012 |
| Antiplatelet medication |  |  |  | <0.0001 |
| No APT | 41/1535 (2.7) | 185/2726 (6.8) | 116/871 (13.3) |  |
| SAPT | 1188/1535 (77.4) | 1923/2726 (70.5) | 557/871 (63.9) |  |
| DAPT | 306/1535 (19.9) | 618/2726 (22.7) | 198/871 (22.7) |  |
| Anticoagulant | 30/1535 (2.0) | 189/2726 (6.9) | 167/871 (19.2) | <0.0001 |
| Beta-blocker | 1188/1535 (77.4) | 2197/2726 (80.6) | 693/871 (79.6) | 0.046 |
| Diuretic | 259/1535 (16.9) | 751/2726 (27.5) | 454/871 (52.1) | <0.0001 |
| Statin | 1433/1535 (93.4) | 2486/2726 (91.2) | 776/871 (89.1) | 0.0011 |

Data are presented as n/N (%) unless otherwise specified.

ACE, angiotensin-converting enzyme; APT, antiplatelet therapy; ARB, angiotensin receptor blocker; BMI, body mass index; bpm, beats per minute; CABG, coronary artery bypass graft; CHF, congestive heart failure; CKD, chronic kidney disease; COPD, chronic obstructive pulmonary disease; DAPT, dual antiplatelet therapy; DBP, diastolic blood pressure; MI, myocardial infarction; NSTEMI, non-ST-segment elevation MI; PCI, percutaneous coronary intervention; PVD, peripheral vascular disease; SAPT, single antiplatelet therapy; SBP, systolic blood pressure; STEMI, ST-segment elevation MI.

**Table S4.** Number of comorbid conditions according to age and functional impairment

A: Age and multimorbidity

| **Age group (years)** | **Number of comorbid conditions** | | | | | | |
| --- | --- | --- | --- | --- | --- | --- | --- |
|  | 0 | 1 | 2 | 3 | 4 | 5 or more | Mean number of comorbid conditions |
| 65–74 | 1119 (32.0) | 1228 (35.1) | 653 (18.7) | 309 (8.8) | 114 (3.3) | 78 (2.2) | 1.2 |
| 75 or older | 416 (25.5) | 497 (30.5) | 348 (21.3) | 195 (12.0) | 111 (6.8) | 64 (3.9) | 1.6 |

B: Functional impairment* and multimorbidity

| **Functional impairment** | **Number of comorbid conditions** | | | | | | |
| --- | --- | --- | --- | --- | --- | --- | --- |
|  | 0 | 1 | 2 | 3 | 4 | 5 | Mean number of comorbid conditions |
| None | 1256 (37.6) | 1163 (34.8) | 580 (17.4) | 231 (6.9) | 68 (2.0) | 40 (1.2) | 1.0 |
| Mild | 181 (19.2) | 319 (33.8) | 221 (23.4) | 130 (13.8) | 66 (7.0) | 26 (2.8) | 1.6 |
| Moderate | 65 (11.9) | 166 (30.3) | 133 (24.3) | 88 (16.1) | 52 (9.5) | 43 (7.9) | 2.0 |
| Severe | 22 (7.9) | 69 (24.6) | 64 (22.9) | 54 (19.3) | 38 (13.6) | 33 (11.8) | 2.4 |

C: Age and functional impairment

| **Age group (years)** | **Functional summary score** | | | | |
| --- | --- | --- | --- | --- | --- |
|  | 0 | 1 | 2 | 3 | Mean functional score |
| 65–74 | 2473 (71.0) | 564 (16.2) | 308 (8.8) | 139 (4.0) | 0.5 |
| 75 or older | 865 (53.3) | 379 (23.3) | 239 (14.7) | 141 (8.7) | 0.8 |

*Created by adding each of the three EQ-5D components (mobility, self-care, and usual activities) scored as 0 (no problems) or 1 (some or severe problems). Data are presented as n (%) unless otherwise specified. p<0.001 for each of the pairwise comparisons.

EQ-5D, five-dimension EuroQol
